# The impact of a nursing spiritual care module on nursing competence: an experimental design

**DOI:** 10.1186/s12904-024-01356-z

**Published:** 2024-01-22

**Authors:** Ali H. Abusafia, Adam Mahmoud Salameh Khraisat, Ola K. Tableb, Khalid Al-Mugheed, Amany Anwar Alabdullah, Sally Mohammed Farghaly Abdelaliem

**Affiliations:** 1 Universiti Sains Malaysia, Penang, Malaysia; 2 Higher Colleges of Technology, Dubai, United Arab Emirates; 3 Riyadh Elm University, Riyadh, Saudi Arabia; 4 Princess Nourah bint Abdulrahman University, Riyadh, Saudi Arabia

**Keywords:** Spiritual care, Competence, Effectiveness, Nurses, Randomized control study

## Abstract

**Purpose:**

This study aimed to assess the impact of the Nursing Spiritual Care Module on the competence of nurses in providing spiritual care in the context of Malaysia.

**Method:**

This study employed an experimental design and involved a total of 122 nurses, with 59 in the experimental group and 63 in the control group. Participants were selected from palliative care wards associated with Hospital Universiti Sains Malaysia. Nurses in the experimental group underwent a two-week educational module on nursing spiritual care, while nurses in the control group attended a single lecture on spiritual care provided by the hospital.

**Results:**

The results indicated no significant differences in sociodemographic characteristics between the two groups. A significant difference in spiritual care competence within the intervention group and the control group over time (*p*-value = 0.001), between the two groups (*p*-value = 0.038), and in the interaction between time and group (*p*-value = 0.001).

**Conclusion:**

The Nursing Spiritual Care Module is crucial in aiding nurses and healthcare professionals in cultivating the appropriate and wholesome attitudes and practices necessary to address the spiritual needs of patients.

**Supplementary Information:**

The online version contains supplementary material available at 10.1186/s12904-024-01356-z.

## Introduction

Spiritual care is an integral and essential component of holistic nursing care, a concept embraced and endorsed by the World Health Organization (WHO) since 1998 [1, 2]. The WHO defines “Health is a state of complete physical, mental, and social well-being and not merely the absence of disease or infirmity” [3]. Consequently, spiritual care has been linked to favorable outcomes such as enhanced patient resilience, decreased pain, stress, and negative emotions [4], as well as a reduced risk of depression and suicide [5]. Patients who get spiritual care also frequently express better satisfaction with both medical and nursing treatments [6].

As a consequence, nurses are responsible for addressing patients’ spiritual needs and gaining the information necessary to properly provide spiritual care while maintaining strong patient relationships [7]. It has been demonstrated that patients’ wellbeing is strongly impacted by unsatisfied spiritual demands [8], weakening their sense of inner peace, maybe lowering their quality of life, possibly raising their risk of depression [9]. However, many nurses lack adequate training in providing spiritual care, and formal nursing education often neglects the dimension of spirituality. Additionally, the assessment of spiritual needs is not consistently conducted by nursing staff [10].

Thus, healthcare professionals require formal education to prepare them for delivering spiritual care. The expertise of nurses is invaluable in upholding the quality of healthcare provided to patients in hospitals, which is a key factor in enhancing hospital quality [11]. Consequently, competence can be defined as a combination of qualities and attributes that enable optimal performance [12].

Spiritual care competence encompasses a set of skills, knowledge, and attitudes utilized in the nursing process [13]. These competencies come into play within the nursing process context, which involves establishing healing connections with patients, being available to them, offering undivided attention, demonstrating empathy, and accommodating the religious needs of patients with specific beliefs [14]. The ongoing initiative involves providing specialized training in spiritual care for nurses, aiming to enhance their competencies in this area [15, 16]. The primary objective is to assess the impact of such training on nurses’ ability to deliver spiritual care and, consequently, to evaluate its influence on caregivers’ overall capacity to provide effective spiritual support.

## Materials and methods

### Study design and setting

This experimental study was conducted as a randomized controlled trial at Hospital Universiti Sains Malaysia (HUSM), is a teaching hospital associated with Universiti Sains Malaysia (USM) located in the northeast region of Peninsular Malaysia. It serves as a tertiary medical center and plays a crucial role in medical education, research, and healthcare services.

### Participants

The study population encompassed all staff nurses engaged in delivering palliative care services at HUSM. There was a total of 134 staff nurses providing palliative care services across four departments, namely Oncology, Nephrology, Cardiology, and Neurology. Palliative care serves as a critical resource for individuals grappling with severe illnesses such as cancer, kidney failure, heart disease, chronic obstructive pulmonary disease, dementia, Parkinson’s disease, and various other conditions [17, 18].

**Fig. 1:**
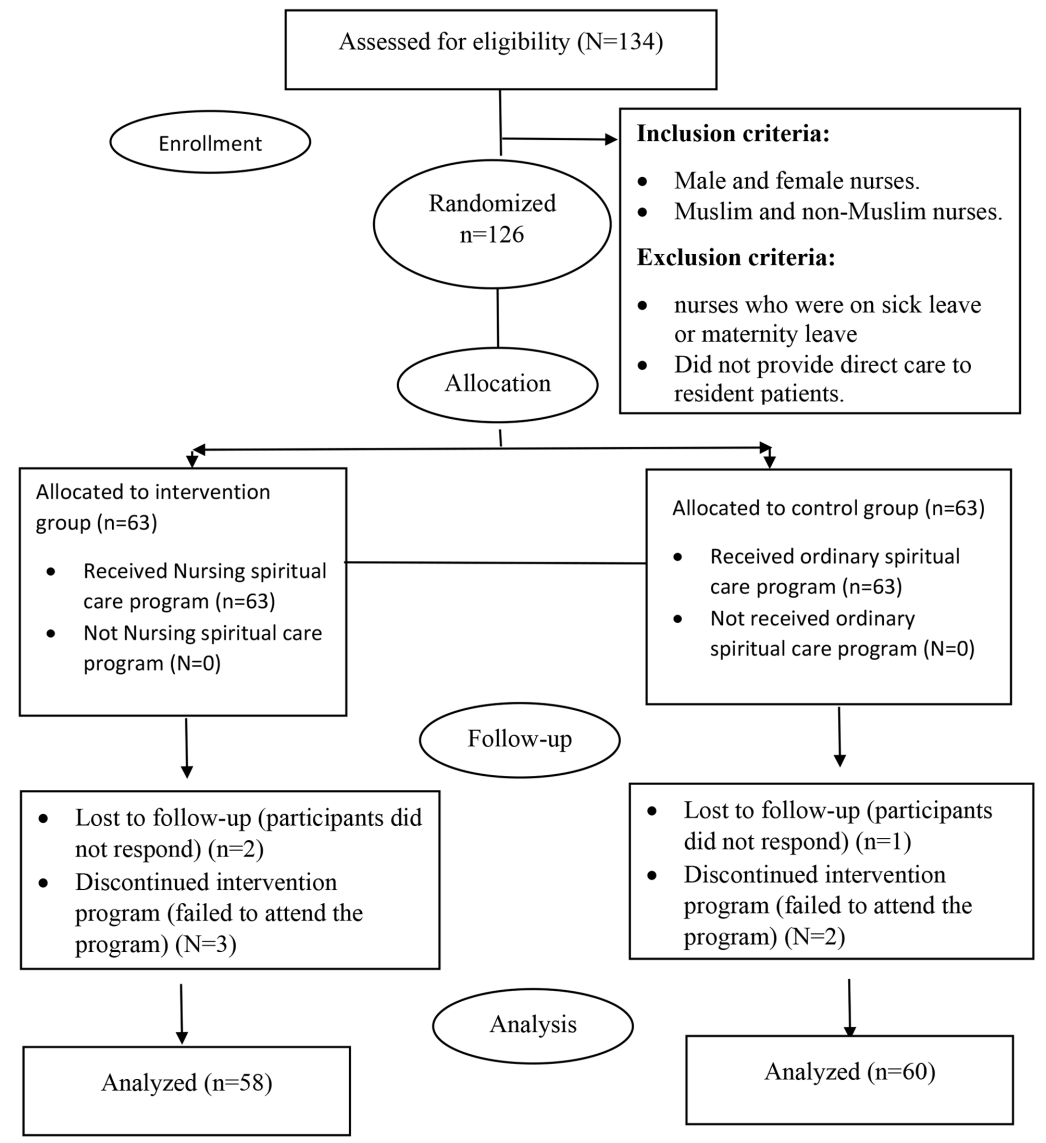
A flow diagram of the study protocol

Inclusion criteria comprised the following: (i) registered male and female nurses actively employed in the cardiology, oncology, nephrology, and neurology departments, and (ii) encompassed both Muslim and non-Muslim nurses. Conversely, exclusion criteria were as follows: (i) Staff nurses on medical or maternity leave, and (ii) Staff nurses not directly involved in providing care to resident patients (see Fig. 1).

### Sample size

The sample size was determined using G*Power 3.0.10 software, with the following parameters: a two-tailed t-test, a medium effect size of d = 0.5, a significance level (α) of 0.05, and a power level (1 - β) of 0.95. Initially, a minimum of 54 participants per group was calculated. To account for potential dropouts during the study, an additional 15% was added, resulting in a final sample size of 63 participants per group (54 / (1–0.15)). Consequently, 126 nurses were required sample size, equally divided between the intervention and control groups, with each group containing 63 participants. To distribute the estimated participants across each department using a sampling fraction (F = n/N), a sampling fraction of 0.94 was applied. In literature reviews, as a general rule, a sample size of 60 participants in each group are considered sufficient for this type of study [19, 20].

### Randomization

This study employed a computer-generated simple randomization method to randomly allocate the palliative care departments into either the intervention or control group. Staff nurses who successfully completed the pre-test questionnaires were randomly assigned to one of two groups based on their department, resulting in 63 participants in the intervention group and 63 participants in the control group.

### Blinded

Participants were kept blind to the allocation and remained unaware of their group assignment. As pointed out by Schulz and Grimes [21], participants who knew they were assigned to a different treatment group might have altered their expectations, while those receiving the standard treatment might have perceived less improvement and potentially withdrawn from the study. Therefore, implementing a blinding procedure in the trial is crucial to mitigate any potential bias in participants’ psychological or physical responses [21].

### Intervention

The spiritual care nursing program consisted of three sessions (refer to Table 1.), conducted over a span of 9 days, with one session held every three days and lasting three hours each. The scheduling of these sessions was coordinated in consultation with the Head of Nurses to avoid conflicts with regular hospital nursing shifts. The three sessions encompassed: (1) an introduction to spiritual care and self-awareness improvement, (2) the assessment of patients’ spiritual needs, and (3) the implementation of nursing spiritual care. This module underwent validation by academic experts from the School of Health (Multidisciplinary Unit and Nursing Program) and the School of Medical Sciences (Medical Education Unit) at Universiti Sains Malaysia (USM), as well as the Islamic Center at HUSM. Additionally, face validity was assessed through a group discussion involving 20 orthopedic nurses with over 10 years of experience, who evaluated the module for clarity, language, applicability, and content flow.

**Table 1:** Structure of nursing spiritual care module (NSCM)

No.	Session	Objectives
1.	Introduction to spiritual care	• To define the concepts of spirituality and spiritual care. • To understand the Nurses’ Role in Spiritual Care • To appreciate the importance of spiritual care for patients. • To discuss nurses’ relationship and attitude towards patients. • To improve and increase spiritual self-awareness.
2.	Assessing the spiritual needs of patients	• To identify the concept of spiritual health, need and distress. • To describe the purpose of conducting a spiritual assessment. • To understand how patients can express his/her spiritual need • To describe how to assess patients’ spiritual need. • To discuss what is the essential skills the nurses should have, to identify the patient’s spiritual care needs.
3.	Spiritual care intervention	• To describe the categories of spiritual care intervention • To understand how to provide spiritual care. • To discuss the importance of therapeutic communication with patients. • To explain the importance of documentation on patients’ spiritual needs. • To explaining how and when nurses can referral the patients to the specialists.

### Ethical consideration

Ethical approval for this study was obtained from the Human Research Ethics Committee of USM prior to its commencement, with a study protocol assigned the code USM/JEPeM/18,080,366. The study’s purpose was explained to the participants, emphasizing the confidentiality and voluntary nature of their involvement. Participants retained the right to discontinue their participation at any point during the study. Informed consent was obtained from each participant who willingly agreed to take part in the study before completing the pre-test questionnaire. Participants were informed of the voluntary nature of their participation, and Continuing Professional Development (CPD) points were provided to them by the Islamic Center at HUSM.

### Data collection

The data for this study was collected through the administration of well-structured and self-administered questionnaires to the respondents. These questionnaires were divided into two parts. The first part focused on sociodemographic factors, encompassing gender, age, marital status, years of experience, level of education, and participation in spiritual care programs, workshops, or training.

The second part involved the use of the self-assessment Spiritual Care Competence Scale Malay version (SCCS-M) (15). The SCCS-M comprises 27 items across six domains, employing a Likert Scale with a 5-point rating scale (1 = strongly disagreed, 5 = strongly agreed). Scores on this scale ranged from 27 to 135, with higher scores indicating a greater level of competence among nurses. The translation into Malay was carried out by the Language Center of USM. The researcher assessed the validity and reliability of the Malay version of the spiritual care competence scale and administered it both before and after the educational program. The Cronbach’s alpha value for the total SCCS-M score was 0.926 [22].

Data was collected twice: initially, the SCCS-M pre-test was administered to both groups before they began the educational program. Subsequently, one month after completing the program, the post-test was administered using the same SCCS-M questionnaire. Campbell and Stanley, recommend that post-tests be administered one month, six months, and one year after the pre-test. Each head of the nurses’ department assisted the researcher in collecting the data [23]. On average, it took approximately 15–20 min to complete the self-administered SCCS-M questionnaire.

### Data analysis

The collected data was analyzed using the Statistical Package for Social Sciences (SPSS) software version 24. A level of significance (*P* < 0.05) was considered in all statistical tests. Descriptive statistics, including frequency, percentage, mean, and standard deviation (SD), were employed to assess the pre/post-test spiritual care competence among nurses in both the control and intervention groups. Two repeated-measures ANOVA was utilized to investigate the time effect, between-group differences, and the interaction between group and time on the mean scores of spiritual care competence.

## Results

A total of 118 respondents (58 participants in the intervention group and 60 participants in the control group) were enrolled in this study. For the intervention group (*n* = 58) the majority of participants 53 (91.4%) were female, 58 (98.3%) Malay and Muslim, 49 (84.5%) were married, 54 (93.1%) were at diploma education level, and 39 (67.2%) did not attend any spiritual care program/workshop before. The average age of participants was 33.38, with an SD of 5.818. Meanwhile, the average experience of the participants was 10.28, with an SD of 5.997. While for the control group (*n* = 60), the majority of participants 52 (86.7%) were female, 58 (96.6%) Malay and Muslim, 48 (80%) were married, 51 (85%) were at diploma education level, and 36 (60%) did not attend any spiritual care program/workshop before. The average age of participants was 34.48, with an SD of 7.027. Meanwhile, the average experience of participants was 10.92, with an SD of 6.884 (see Table 2).

**Table 2:** Sociodemographic variable

Factors	Intervention group (*N* = 58)		Control group (*N* = 60)	
	Mean (Stander Deviation)	Frequency (%)	Mean (Stander Deviation)	Frequency (%)
**Gender**				
Male		5 (8.6)		8 (13.3)
Female		53 (91.4)		52 (86.7)
Age	33.38 (5.818)		34.48 (7.027)	
**Marital status**				
Single		9 (15.5)		12 (20.0)
Married		49 (84.5)		48 (80.0)
**Education level**				
Diploma		54 (93.1)		51 (85.0)
Bachelor		4 (6.9)		9 (15.0)
**Religion**				
Muslim		57 (98.3)		58 (96.7)
Non-Muslim		1 (1.7)		2 (3.3)
**Nationality**				
Malay		57 (98.3)		58 (96.7)
Non-Malay		1 (1.7)		2 (3.3)
Experience years	10.28 (5.997)		10.92 (6.884)	
**Attendance previous workshop**				
Yes		19 (32.8)		24 (40.0)
No		39 (67.2)		36 (60.0)

The total sample size included 58 participants in the intervention group and 60 participants in the control group. Table 3 presents the distribution of spiritual care competence scores before and after the intervention for both control and intervention groups.

**Table 3:** Assess the level of spiritual care competence pre and post-test among nurses in hospital USM.

Groups (Number participants)	Pre SC test		Post SC test	
	N (%)^1^	Mean (Stander Deviation)^2^	N (%)^1^	Mean (Stander Deviation)^2^
**Intervention group (** ***n*** ** = 58)**		100.328 (8.480)		105.897 (7.462)
Lower 64	-		-	
Average 64–98	17 (29.30)		9 (15.5)	
Above 98	41 (70.70)		49 (84.5)	
**Control group (** ***n*** ** = 60)**		100.067 (8.754)		101.183 (7.172)
Lower 64	-		-	
Average 64–98	22 (36.70)		18 (30.00)	
Above 98	38 (63.30)		42 (70.00)	

^1^ Check the percentage of spiritual care competence in each level

^2^ Check the mean and stander deviation (SD) of spiritual care competence score

In the pre-test, 70.7% (*n* = 41) of participants in the intervention group reported having a high spiritual care competence score, with an average of 29.3% (*n* = 17) reporting an average score. Meanwhile, in the control group, 63.3% (*n* = 83) of participants reported having a high score (above 98), with an average of 36.7% (*n* = 22) having an average score.

In the post-test, 84.5% (*n* = 49) of participants in the intervention group reported having a high spiritual care competence score, and 15.5% (*n* = 9) reported an average score. Conversely, in the control group, 70% (*n* = 42) reported having a high score (above 98), and 30% (*n* = 18) had an average score.

Furthermore, the mean spiritual care competence score in the intervention group was 100.328 with a standard deviation (SD) of 8.480 in the pre-test, and 105.897 with an SD of 7.462 in the post-test. In contrast, the mean spiritual care competence score in the control group was 100.067 with an SD of 8.754 in the pre-test, and 101.183 with an SD of 7.172 in the post-test (see Table 3).

The evaluation of the effectiveness of the spiritual care nursing intervention module was conducted by comparing the intervention and control groups based on their effects over time, between-group differences, and time*group interactions.

With regard to the time impact, the analysis showed a significant difference in the intervention group’s mean score for spiritual care, with F (1, 116) = 14.56 and *P* = 0.001 in the study. Only the intervention group showed a significant difference in the pre-posttest according to pairwise comparisons with a 95% confidence interval adjustment and the Bonferroni correction (mean difference = 5.569, 95% CI: 2.818–8.320; *p* = 0.001). In contrast, there was no discernible difference in the outcomes for the control group in the pre-posttest (mean difference = 1.117, 95% CI: -1.078-3.312; *p* = 0.313).

The analysis of between-group effects demonstrated a significant difference between the intervention and control groups (*p* value = 0.037) in the post-test spiritual care competence scores. Specifically, the mean score for spiritual care competence in the intervention group was higher than that in the control group (see Table 4). This result indicates that the intervention and control groups exhibited different levels of spiritual care competence among nurses.

**Table 4:** Summary of two-way repeated measure ANOVA for spiritual care competence score (within and between subject effects)

Source of variance	Comparison of SCCS	Mean score	95%CI	*P*-value
Within Time (Pre-Post test)	Intervention group	5.569*	2.818–8.320	**0.001** ^**a**^
	Control group	1.117*	-1.078-3.312	0.313^a^
Between group	Intervention group	103.112	101.442-104.782	**0.037** ^**b**^
	Control group	100.625	98.983-102.267	
Time*group Interaction (SCCS Pretest)	Intervention group	100.328	98.087-102.568	0.870^c^
	Control group	100.067	97.864–102.270	
Time*group Interaction (SCCS Posttest)	Intervention group	105.897	103.994-107.799	**0.001** ^**c**^
	Control group	101.183	99.313-103.054	

*MD = mean difference

^a)^ Repeated measures ANOVA within-group analysis was applied followed by pairwise comparison with 95% confidence interval adjustment by Bonferroni correction. MD = mean difference

^b)^ Repeated measures ANOVA between-group analyses were applied; F-stat (df) = 4.423 (1); Level of significance was set at 0.05 (two-tailed)

^c)^ Repeated measures ANOVA between group analysis with regard to time was applied followed by pairwise comparison with 95% confidence interval adjustment by Bonferroni correction. Assumption of normality, homogeneity of variances and compound symmetry were checked and were fulfilled

The analysis of the interaction between time and group revealed a significant difference in the post-test spiritual care competence of the intervention group, as indicated by the estimated marginal means for spiritual care competence (*P* = 0.001, see Table 4). However, the estimated marginal means for spiritual care competence in the pre-intervention group yielded a non-significant result. These findings suggest that the impact of group conditions on nurses’ competence in the field of spiritual care changed over the course of a month.

**Fig. 2:**
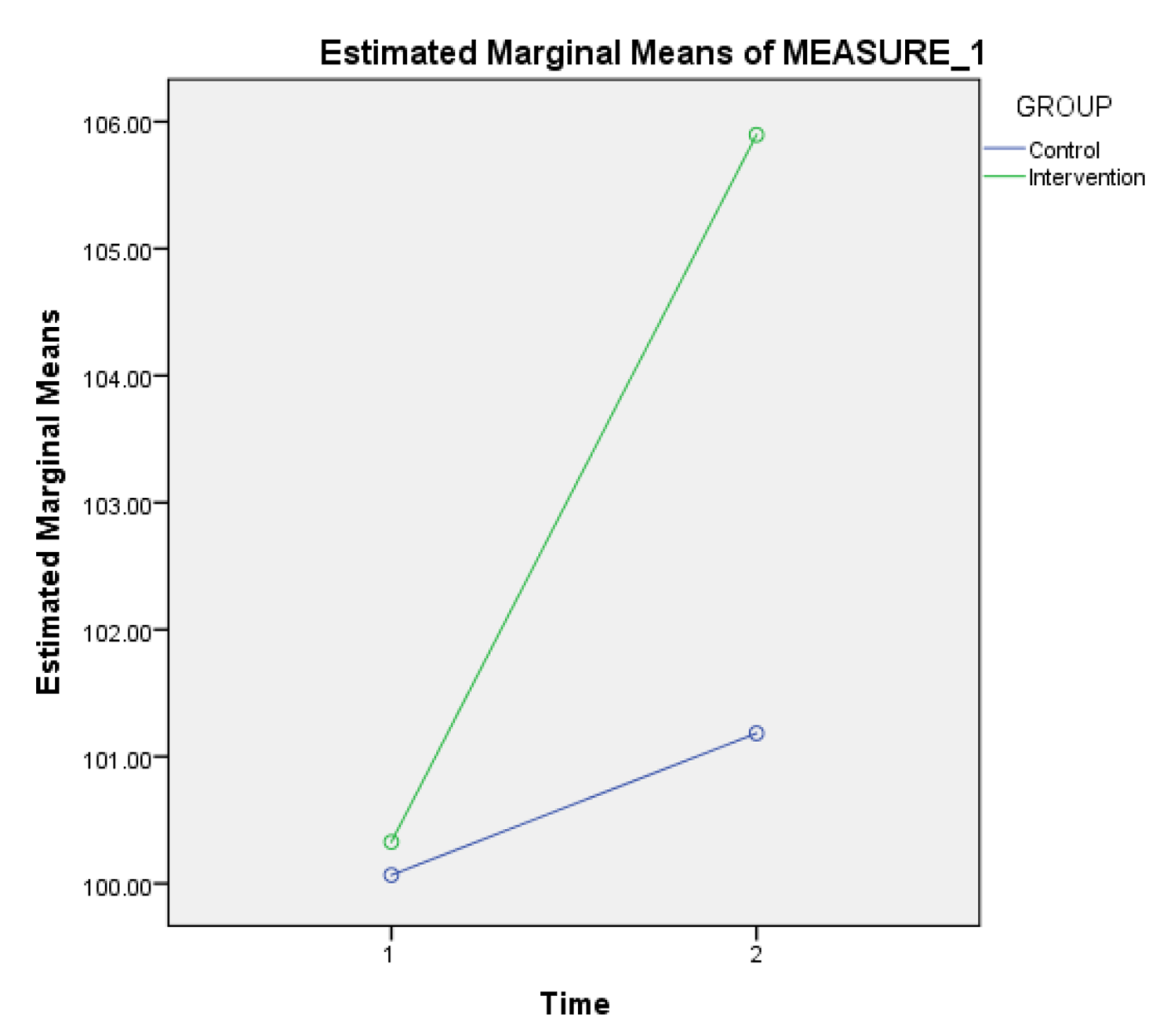
The adjusted mean (estimated marginal means) of spiritual care competence scores for pre and post interventions

Figure 2 displays the profile plot of the adjusted mean (estimated marginal mean) of the pre-test and post-test spiritual care competence scores. In the pre-test, scores were nearly equivalent for both groups. However, following the intervention and post-test, the spiritual care competence score for the intervention group increased by 4.714 compared to the control group. This outcome suggests a significant interaction effect between the two independent variables: group and time. Additionally, it indicates a more pronounced difference between the two groups in the post-test compared to the pre-test.

## Discussion

According to the study’s findings, there were no appreciable sociodemographic differences between the nurses in the intervention and control groups. This implies that the participant distribution in these groups was efficient and similar. This study had a high response rate of 96.82%, which is comparable to a study on nursing students in the Netherlands, which had a response rate of 100% [24]. In different study among American nursing staff (93%) [25]. It was, nevertheless, a little higher than a comparable study done among nurses in the Netherlands (86%) [26], and an online research study done with participants from the Association of Pediatric Hematology/Oncology Nurses (APHON) in the United States, Canada, and other nations (56%) [27]. These response rates highlight nurses’ interest in acquiring knowledge and improving their nursing skills through educational programs and other means. Furthermore, the Malaysia Nursing Board encourages nurses to engage in Continuing Professional Education (CPE) activities to enhance their knowledge, skills, and attitudes [28].

In this study, the results of the pre-test indicated that the mean score of spiritual care competence in the intervention group was 100.328 with a standard deviation (SD) of 8.480, while in the control group, the mean was 100.067, with an SD of 8.754. These findings are somewhat similar to an online study conducted by APHON, which reported a mean nursing competence score of 98.30 with an SD of 14.05 [27]. However, they differ from previous studies in the United States, where the mean nursing competence score was 71.96 (SD = 16.49) [28].

The higher level of spiritual care competence among nurses in this study may be attributed to the fact that the majority of them are Muslim. In Islam, Muslims place a high value on the Holy Quran as a source of spiritual guidance and solace. The Islamic Center in HUSM also plays a significant role in enhancing nurses’ awareness and knowledge of spiritual care through lectures and courses. After one month of applying the nursing spiritual care education program, the intervention group showed an improved mean score (Mean = 105.897, SD = 7.462) of nursing competence compared to the control group (Mean = 101.183, SD = 7.172), which remained relatively consistent with the pre-test results. This result aligns with previous studies that demonstrated better improvement in the intervention group compared to the control group, highlighting the importance of education and specialized training in spiritual care for nursing staff [27, 29, 30]. Many studies have indicated that nurses often lack sufficient knowledge in the field of spiritual care, which contributes to the neglect of spiritual care and patients’ spiritual needs [31–33]. Therefore, the inclusion of a nursing spiritual care education module in this study serves as a means to address this knowledge gap and emphasize the significance of spiritual care in nursing practice.

Considering the time effect, a significant difference was observed between the pre- and post-tests in the intervention group (*P* value = 0.001), indicating that the spiritual care competence of nurses in this group improved after one month of participating in the educational program. This outcome is consistent with a study conducted among nurses by Kim, which where the mean score of the intervention group increased from 68.6 to 83.9 after seven weeks of the educational program [34]. In a subsequent research including nursing students, Chung and Eun, discovered a highly significant difference (p 0.001) in the mean score of the intervention group, which increased significantly from 78 to 97 following training [35]. Similar results were found in research of clinical nurses in a Chinese hospital for cancer treatment, where the mean score of the study group significantly increased from 79.24 to 110.84 with a *P* value of 0.05 [30].

These substantial consequences were especially noticeable a month after the training program began, indicating that it takes time to put spiritual care ideas into practice. Giving nurses the time, they need to put their new abilities to use is crucial for achieving this. Additionally, it’s important to spend time with patients while providing spiritual care since it helps to get the best results. Previous studies that stressed the requirement for healthcare professionals to provide enough time for spiritual care in their nursing plans lend credence to this [36].

The results of this study show that the nursing spiritual care module had a beneficial effect on nurses in the intervention group, who were more competent as a result of taking part in the educational program. The design of the nursing spiritual care program, which emphasizes improving nurses’ spirituality and providing them with the strategies and resources they need to give spiritual care to patients, should be credited with these advances. According to Burkhart and Hogan, notion of spiritual care, the competency of nurses is positively impacted when they become more spiritually aware and learn how to improve their spiritual well-being, enabling them to better address the spiritual requirements of patients [37].

The concentration on individual spirituality in the nurses’ spiritual care module in addition to the inclusion of the nursing treatment plan as a crucial element of their work are believed to be responsible for the differences between the intervention and control groups that were noticed. Through this program, nurses are trained to comprehend the essence of spiritual care and to employ the five-step nursing process (comprising assessment, diagnosis, planning, implementation, and evaluation) in the context of spiritual care. It is imperative to underscore the significance of utilizing the nursing process to discern and execute the most effective interventions for enhancing patient well-being. The more extensively we apply the nursing process, the more targeted, efficient, transparent, and evidence-based nursing interventions will be [38].

In recent years, there has been a growing body of research focused on spiritual care worldwide. Nevertheless, this study stands out as the first to develop a nursing spiritual care module in Malaysia. The primary objective of this educational program is to foster nurses’ understanding of the methodologies employed in delivering spiritual care to patients. It also aims to bolster and augment their knowledge, skills, and attitudes pertaining to spiritual care. The findings of this study corroborate the practicality and feasibility of the nursing spiritual care module in enhancing the competence of nurses in the realm of spiritual care. Additionally, this study represents the pioneering effort in Malaysia to introduce a spiritual care education program for nurses. Another limitation is the predominantly Muslim and Malay composition of the participant pool, which results in a lack of diversity in terms of religion, culture, and demographics. Future research should encompass a more diverse range of participants to gain a more comprehensive understanding and achieve broader applicability of the outcomes.

## Conclusion

Beginning with the concept of spiritual care competence, this study designed and introduced the nursing spiritual care module to enhance nurses’ proficiency in the realm of spiritual care. The outcomes demonstrate that this module effectively elevates nurses’ competence in providing spiritual care. These results carry valuable implications for nursing staff, nursing students, and other healthcare practitioners, elucidating the efficacy of nursing spiritual care. This, in turn, enables them to offer appropriate spiritual guidance and promote health awareness among both patients and their families.

### Electronic supplementary material

Below is the link to the electronic supplementary material.

Supplementary Material 1

## Data Availability

Data available on request from the authors.
