# Supplementary material for: The impact of a nursing spiritual care module on nursing competence: an experimental design
Source: BMC Palliat Care. 2024 Jan 22;23:21. doi: 10.1186/s12904-024-01356-z (PMC10802070; doi:10.1186/s12904-024-01356-z)
Supplement: Supplementary file 1 — Supplementary Material 1 [file 12904_2024_1356_MOESM1_ESM.pdf]

(Appendix 1)

Spritual Care Competence Scale. For each item, please estimate the level of efficiency by circling the right answer which will show how far you agree or disagree with the statements.

SD(1): Strongly Disagree D (2): Disagree MD(3): Moderately Disagree

MA(4): Moderately Agree) A(5):Agree SA(6):Strongly Agree

	Items	SD	D	MD	MA	A	SA
	**The evaluation and implementation of spiritual care**						
1	I can report verbally orally and/or in writing on the spiritual needs of patient.						
2	I can customize the care with patient's spiritual needs/problems in consultation with patient.						
3	I can customize the care with patient's spiritual needs/problems through multi-disciplinary consultancy sessions.						
4	I can record the nursing component on patient's spiritual care in the nursing plan.						
5	I can record a patient's spiritual functions in writing.						
6	I can record a patient's spiritual functions orally.						
	**Professionalization and improving the quality of spiritual care**						
7	I can contribute to the quality assurance in spiritual care at the nursing ward.						
8	In nursing ward, I can contribute professional development in spiritual care in the nursing ward.						
9	I can identify the problems related to spiritual care during the peer discussion session.						
10	I can train other staff in the field of spiritual care delivery to the patient.						
11	I can make policy recommendation in the aspect of spiritual care to the management in the nursing ward.						
12	I can implement the spiritual care improvement project in nursing ward.						
13	I can provide spiritual care to patient.						
14	I can evaluate the spiritual care that I have delivered during consultation with the patient in one disciplinary/multi-disciplinary team.						
15	I can give information to the patient regarding spiritual facilities in an institutional care (including spiritual care, meditation center, and						

	religion service).						
16	I can help the patient to continue his/her daily spiritual practice (including providing opportunities for rituals, prayers, meditation, reciting bible/Quran, listening to music).						
17	I can care to patient's spirituality for the daily care (e.g.: physical care)						
18	I can refer patient's family to a spiritual advisor/pastor, if they ask me and/or indicate their spiritual needs.						
	**Referral**						
19	I can determine the patient's need for spiritual care to the provider/ care worker/other care discipline effectively.						
20	I can refer patient to the other care worker at the suitable time effectively (e.g. : pastor/the patient's pastor /*imam.*						
21	I know when I need to consult spiritual advisor regarding patient's spiritual care.						
	**Attitude towards the patient's spiritual**						
22	I show unprejudiced respect towards patient's spiritual/ religious beliefs regardless of their spiritual/religious background						
23	I am open to patient's spiritual/ religious beliefs although it differs from my own belief.						
24	I try to not force my spiritual belief/religion to patient.						
25	I am aware of my personal limits when I face with patient's spiritual belief/ religious.						
	**Communication**						
26	I can listen actively to patient's life story relating to their illness/disability.						
27	I have an accepting attitude in my dealings with a patient (e.g.: concerned, sympathetic, inspirational trust and confidence, genuine, empathetic, sensitive, sincere and personal)						
